# Bioinspired Helicoidal Composite Structure Featuring Functionally Graded Variable Ply Pitch

**DOI:** 10.3390/ma14185133

**Published:** 2021-09-07

**Authors:** Michele Meo, Francesco Rizzo, Mark Portus, Fulvio Pinto

**Affiliations:** Material and Structure Centre, Department of Mechanical Engineering, University of Bath, Bath BA2 7AY, UK; f.rizzo@bath.ac.uk (F.R.); mcsp20@bath.ac.uk (M.P.); f.pinto@bath.ac.uk (F.P.)

**Keywords:** impact resistance, residual strength, bioinspiration, helicoidal, composite

## Abstract

Composite laminated materials have been largely implemented in advanced applications due to the high tailorability of their mechanical performance and low weight. However, due to their low resistance against out-of-plane loading, they are prone to generate damage as a consequence of an impact event, leading to the loss of mechanical properties and eventually to the catastrophic failure of the entire structure. In order to overcome this issue, the high tailorability can be exploited to replicate complex biological structures that are naturally optimised to withstand extreme impact loading. Bioinspired helicoidal laminates have been already studied in-depth with good results; however, they have been manufactured by applying a constant pitch rotation between each consecutive ply. This is in contrast to that observed in biological structures where the pitch rotation is not constant along the thickness, but gradually increases from the outer shell to the inner core in order to optimise energy absorption and stress distribution. Based on this concept, Functionally Graded Pitch (FGP) laminated composites were designed and manufactured in order to improve the impact resistance relative to a benchmark laminate, exploiting the tough nature of helicoidal structures with variable rotation angles. To the authors’ knowledge, this is one of the first attempts to fully reproduce the helicoidal arrangement found in nature using a mathematically scaled form of the triangular sequence to define the lamination layup. Samples were subject to three-point bending and tested under Low Velocity Impact (LVI) conditions at 15 J and 25 J impact energies and ultrasonic testing was used to evaluate the damaged area. Flexural After Impact (FAI) tests were used to evaluate the post-impact residual energy to confirm the superior impact resistance offered by these bioinspired structures. Vast improvements in impact behaviour were observed in the FGP laminates over the benchmark, with an average reduction of 41% of the damaged area and an increase in post-impact residual energy of 111%. The absorbed energy was similarly reduced (−44%), and greater mechanical strength (+21%) and elastic energy capacity (+78%) were demonstrated in the three-point bending test.

## 1. Literature Review

Laminated composite materials are used in engineering to take advantage of the high specific strength and stiffness they can provide, in addition to their excellent fatigue and corrosion resistance. A notable disadvantage in many laminated composites, however, is their susceptibility towards out-of-plane loading, with the subsequent generation of damage within the structure, such as delamination, crack and fibre failure. This can greatly affect the performance of the part and could lead to the sudden and catastrophic collapse of the entire system [1]. In order to overcome this issue, several solutions can be found in the literature aiming to increase the impact properties of these materials, including single component modification [2,3,4], hybridisation with metal wires [5], the introduction of non-Newtonian fluids [6] and the use of a polymeric coating as a superficial protective layer [7]. In this context, biological structures, such nacre [8], the cuticle of a Scarabaeidae beetle [9] and the dactyl club of the mantis shrimp [10], constitute a very interesting source of inspiration since they naturally evolved to function as impact-resistant armour and weaponry for protection and hunting. For instance, the nacre structure shows a brick-and-mortar inner configuration in which strain-hardening features are activated during failure [11,12], while the beetle cuticle is organised in a constant pitch helicoidal structure formed by a constant rotation of the layers through the thickness—also known as a “Bouligand” structure [13,14,15]. Extensive research has been focused on the investigation of the behaviour of the helicoidal structure under dynamic conditions, showing improved impact resistance as result of the activation of an additional energy dissipation mechanism called crack twisting [9]. This mechanism promotes the formation of microcracks that follow the helicoidal orientation of the layers, creating a twisted crack front, dissipating a higher amount of energy in a longer crack path without severe detriment to the mechanical properties and/or catastrophic failure [16]. 

The effectiveness in mimicking the crack twisting mechanisms in composites was investigated by Suksangpanya et al. [17], who used 3D printing to create a structure representing a helicoidally laminated composite. Three-point bending tests were then carried out on the structures to investigate the damage mechanisms, resulting in twisting cracks through the matrix with no fracture of the fibres. This research highlights the changing damage mechanisms through the thickness of the composites, which is also dependent on the constituent fibre and matrix properties in addition to the stacking sequence of the laminate. Shang et al. [18] conducted flexural testing on circular helicoidal laminate plates finding improved mechanical performance using a small angle in the helicoidal structures in comparison with cross ply laminates. Ginzburg et al. [19] instead experimentally and numerically evaluated the impact performance of helicoidal laminates using different stacking-up sequences featuring a small pitch angle. The authors observed a reduced damaged area at similar values of absorbed energy, reporting a higher damage tolerance when helicoidal laminates were compared to cross-ply and quasi-isotropic ones. This confirms that the activation of the crack twisting mechanism also for this study case has a less detrimental effect regarding the damage to the residual mechanical properties of the bioinspired laminates in comparison with traditional ones. 

However, even though excellent results have been obtained, these biological structures (i.e., the dactyl club structure of the shrimp mantis) can still offer a precious source of inspiration for bioinspired composites and be a further significant step forward in the development of impact-resistant laminates.

Indeed, past research widely investigated only a partial aspect of the shrimp mantis complex structure, i.e., the simple helicoidal layup in designing bioinspired materials, while only little research has, instead, been completed regarding the full-inspiration and replication of this biological structure to exploit its full potential in preventing impact damage. Indeed, the mantis structure is composed of a complex microgeometry, which includes periodic and striated regions: whilst striated regions—composed of aligned mineral fibres arranged in a circumferential band to avoid lateral expansion of the structure during a strike—aid the structural stability of the dactyl club, previous research has concluded that the periodic region—composed of protein fibres in a laminated periodic pattern—is fundamental in the energy dissipation and impact resistance [20]. Moreover, scanning electron microscopy on the dactyl club has indeed revealed an interesting variation in the inter-ply pitch angles in the periodic region. In particular, the biological structure increases in pitch from 1.6° closest to the impact region to 6.2° at the innermost region of the club in a near-linear manner [9,20]. This small angle variation not only is able to activate the aforementioned crack twisting mechanism seen in the Scarabaeidae beetle cuticle, but also changes the mechanical properties of the laminate along its thickness, creating a Functionally Graded (FG) material [21] showing unique behaviour in terms of dynamic response [22] and failure mechanisms [23], including crack propagation [24] and delamination [25]. Bamboo [26], human bones [27] and alligator osteoderm [28] are only few examples of the numerous FG structures that can be found in nature. These biological structures evolved their configuration towards the satisfaction of specific structural requirements in order to ensure the survival of the entire biological system by tuning the heterogeneous structural parameters across the material’s thickness, such as composition [29,30], arrangement [28,31], dimension [32,33] and orientation [34,35]. This affects the mechanical properties in specific regions of the structure, allowing to optimise the response in function of a determined solicitation (predator’s attack, environmental threat, etc.). Another important characteristic typical of FG materials is the presence of a gradual and smooth variation in the properties of the areas comprised between the functionalised regions in order to reduce the stress localisation in correspondence with their interfaces and maximise the overall performance of the system. 

Focusing the attention on FG materials featuring a functional variation of its components’ orientation, the body armour of Arapaima gigas fish can be considered as one of the possible examples. This structure is composed by lamellae of which the cartilaginous fibrils are tilted by ~35° from the adjacent ones. This maximises the impact and penetration resistance of the system against foreign objects by the activation of crack twisting mechanisms similar to the ones previously described for Bouligand structures. Consequently, as confirmed by [26], it is evident that a connection between the FG material with functional orientation and Bouligand structures can be found considering the latter as a subcategory of FG materials.

Several researchers focused their attention on replicating the unique impact resistance and failure mechanisms of FG biological structures featuring functional orientation in laminated composites. 

This work is focused on the design and development of a bioinspired helicoidal composite characterised by a graded pitch angle that exhibits improved impact behaviour whilst maintaining high mechanical properties. Utilising this variable angle ply replicated from the inner structure of dactyl club of mantis shrimp, smaller angles of rotation were located in proximity of the laminate surface in order to improve the damage tolerance via the activation of enhanced crack twisting mechanisms during an impact event. The mechanical strength, stiffness and impact properties of the laminates were evaluated by experimental testing to assess and prove the potential of these bioinspired Functionally Graded Pitch (FGP) helicoidal structures in advanced applications while ultrasound techniques were used to evaluate the damaged areas and correlate their extent with the residual mechanical properties evaluated using experimental post-impact testing.

## 2. Functionally Graded Pitch (FGP) Laminates

In order to mimic the unique structure of the mantis shrimp and obtain a high-performance bioinspired laminated composite, a deep understanding of the enhanced failure mechanisms of the helicoidal laminates is fundamental to design the stacking sequence of these materials and maximise their potentialities towards impact events. In this section, firstly a systematic analysis of the aforementioned crack twisting mechanism will be illustrated (Section 2.1). Afterwards, the design used in this work will be presented (Section 2.2), with focus on the issues found during the replication of biological structure in the laminated one. In Appendix A, the analysis of these limitations and an adequate solution is presented.

### 2.1. Mechanical Performance

In laminated composites, three main damage typologies can be identified as the main cause of failure: fibre failure, intralaminar fracture (matrix cracking) and interlaminar fracture (delamination). Crack twisting is a failure mechanism generated by the presence of a small ply angle between two different layers of the material based on the principle that cracks tend to propagate along the path that requires the lowest amount of energy to generate new surfaces [36]. It can be explained focusing the attention on the delamination fracture energy and its dependency on the ply orientation. Indeed, even though the critical energy for an intralaminar fracture can be considered almost constant in the function of the ply angle, the critical energy for the interlaminar one strongly depends on the ply angle between two adjacent layers, as reported by several authors [37,38,39]. This concept was experimentally investigated by Kim [40] who reported that the interlaminar fracture toughness can be associated with the mixed mode fracture toughness G, expressed as

(1) $$G=G_{I}+G_{II}$$

where *G_I_* and *G_II_* are the Mode I and Mode II fracture toughness (kJ/m^2^) experimentally evaluated using Mixed Mode Bending (MMB) tests. The values of these two toughness can be then calculated from the experimental data using Equations (2) and (3) [40]:

(2) $$G_{I}=\frac{{\left(F\left(3s-l\right)\right)}^{2}}{16bl^{2}E_{11f}I}{\left(a+h\right)}^{2}\;$$

(3) $$G_{II}=\frac{3{\left(F\left(c+l\right)\right)}^{2}}{64bl^{2}E_{11f}I}{\left(a+0.42h\right)}^{2}$$

where *F* is the maximum force recorded before the load drop, a and s are the initial delamination and span lengths, respectively; *l, h* and *b* are half of the length, thickness and width of the sample, respectively; and *E_11f_* is the flexural elastic modulus in the direction of the fibres while *I* is the area moment of inertia of one of the delaminated portions. Several stacking-up configurations at different ply angles were tested in this work, reporting accurate results regarding the dependency of *G* on the difference in angle orientation between two adjacent plies. Afterwards, the relationship between *G_I_* and *G_II_* and the delamination fracture toughness *G_c_* can be obtained by using the following semi-empirical formulation:

(4) $$G_{c}=A+BG_{II}G^{m}$$

where *A*, *B* and *m* are the coefficients extrapolated via the non-linear regression (power law) of the experimental data. Thus, following this approach, it is possible to experimentally correlate the ply orientation used in the stacking-up of the laminate to the *G_c_* value. Results clearly showed that that delamination fracture toughness is inversely proportional to the angle ply. In particular, the authors showed that two adjacent plies with a mismatch angle of 90° have a reduction of ~50% in terms of fracture toughness in comparison with a mismatch angle of 0°. Another result reported in this work is that the *G_c_* dependency on the ply angle is also strongly influenced by the loading conditions applied to the laminate: the closer to the pure Mode II loading, the stronger the dependency of *G_c_* on the ply orientation. Following the same concept, Anderson et al. [38] also investigated the relationship between *G_c_* and ply orientation and proposed an equation to approximate the G_IIc_ value from the ply angle *θ* and the value of fracture toughness with *θ* = 0° (*G_II0_*). They concluded that it is possible to predict the Mode II interlaminar fracture toughness by using the formula

(5) $$G_{IIc}=G_{II0}+Btan\theta$$

where *B* is a coefficient that takes into account the additional shear stress contribution. Considering the loading conditions found in impact events, the main cause of failure within the composite structure can be related to the shear stress [41,42,43] localised within a structure during the dynamic loading. Consequently, it is possible to assume that Mode II failure is dominant and high dependency from the ply angle is expected for the *G_c_* value in this loading condition. 

**Figure 1:**
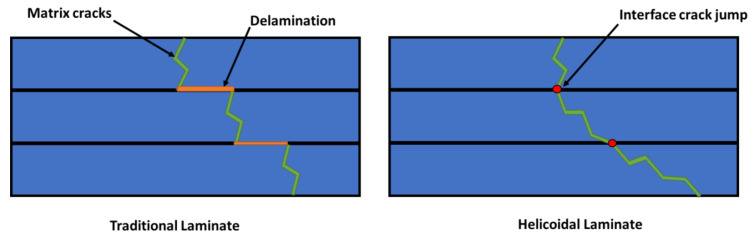
Illustration of the crack twisting mechanisms of a helicoidal laminate’s failure.

Following these considerations and analysing the effect of ply angle on the failure behaviour of composite materials, it is possible to observe that the smaller the angle used between two consecutive plies, the higher the delamination fracture toughness. On the other hand, increasing the ply angle, the delamination fracture toughness decreases. Hence, when the angle between two adjacent plies changes by a large quantity, the crack prefers to propagate along the interlayer interface instead of generating intralaminar cracks since the fracture energy required for creating delamination is lower. On the other hand, when smaller angles are used, the energy required to propagate the damage along the interface is higher and, consequently, it is energy-wise easier for the crack to propagate across the matrix of the layer following the ply angle and “jump” across the interface between layers [10]. A schematisation of this concept is reported in Figure 1, where an helicoidal laminate is compared to a traditional one showing their differences in terms of failure mechanisms.

This was confirmed by Liu et al. [44] in their experimental study on helicoidal laminates, in which they report that larger delaminated areas are generated within laminates with a large ply angle since the interlaminar shear strength lowers its value accordingly with the increase in angle. Consequently, the use of helicoidal configurations with a small ply angle reduces the extent and number of delaminated areas if compared with traditional ones, allowing the system to tolerate a higher contact force while dissipating a similar amount of impact energy [19,45]. Another aspect of this behaviour is also the generation of subcritical damaged areas [16] within the laminate, a typology of damage that shows reduced effects on the performance of the laminate with no sign of critical failure and load drops. This enables the structure to absorb a higher amount of energy and tolerate a higher contact force than traditional laminates. 

While it is clear that the helicoidal configuration improves the out-of-plane properties, it is important to notice that a decrease in out-of-plane stiffness is observed when a small ply angle is used due to the reduced number of plies oriented along the principal directions of the laminate [46]. In particular, by increasing the ply angle, it is possible to increase the stiffness of the material, but the effectiveness of the crack twisting mechanisms is reduced. A compromise between in-plane (stiffness) and out-of-plane (crack twisting) is then necessary. 

**Figure 2:**
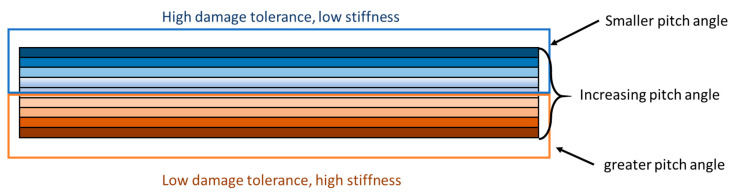
Schematisation of the mechanical behaviour of FGP laminates.

This compromise is found in the use of an FGP angle across the thickness’s direction of the laminate, as seen in FG materials (Figure 2). Indeed, using a small ply angle in the proximity of the impact event, it is possible to promote crack twisting mechanisms and, thus, enhance the damage tolerance of the laminate via the creation of sub-critical damage. When an impact happens, the crack and delamination opening initiates and firstly propagates in these regions, maximising the effect of the crack twisting mechanisms and dissipating most of the energy received from the impact event. By increasing the distance from the impact event, since most of the impact energy has been already absorbed in the upper portion of the laminate and, consequently, no significant delaminated areas can be generated in this portion, the increased pitch angle has the function to limit the in-plane stiffness reduction.

Based on these considerations, the use of FGP represents a cutting-edge solution for the creation of high-performance bioinspired composites to satisfy the requirements of advanced application in which high load and damage tolerances are fundamental for the safety and reliability of the primary load-bearing structures.

### 2.2. Design Description

Due to the nature of the thin laminae in biological composites and the gradual development of these complex biological structures across the growing process of the organism, it is not practically possible to manufacture a fully accurate biomimetic composite from synthetic CFRP material. This is due to the intrinsic nature of composites manufacturing that requires the use of temperature and pressure that can create distortion or geometrical defects if not carefully carried out. Consequently, to enable the manufacturing of helicoidal composite structures with a reasonable thickness that closely mimic the dactyl club structure of a mantis shrimp, the pitch angle change of the composite was completed over one full rotation of constituent laminae—a notable difference to the biological structure, which completes this change over many complete rotations [47]. The ply angle was increased along the laminate’s thickness following a mathematically scaled triangular sequence as observed in the literature [48]. The formula used to define the sequence is reported in Equation (6).

(6) $$c\overset{n}{\underset{k=1}{\sum }}1+2+3+5+\ldots =c\frac{n\left(n+1\right)}{2}$$

where *c* is the scaling coefficient.

**Figure 3:**
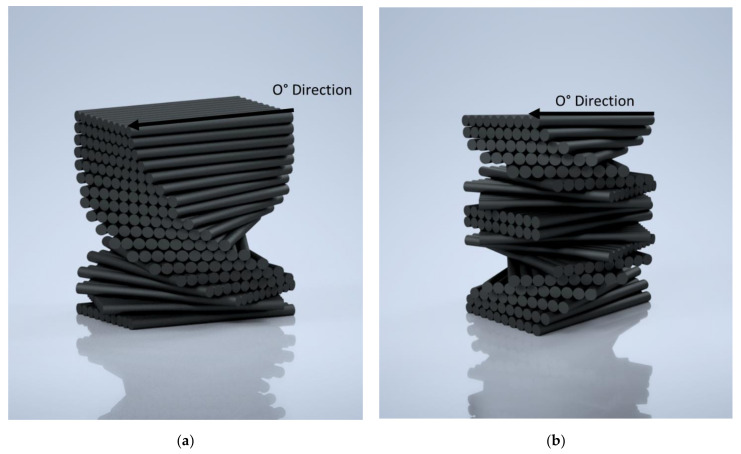
Layout of the designed helicoidal structures: (**a**) asymmetric (FGPA) and (**b**) symmetric (FGPS).

Based on this formula, the Functionally Graded Asymmetric (FGPA) lamination sequence (Figure 3a) was designed with an initial small ply angle (~1.2–1.8°). However, due to the asymmetrical layup sequence used during the manufacturing process, the presence of thermal warpage (Appendix A) can represent a technical and geometrical issue for this structure. 

Thus, a Functionally Graded Pitch Symmetric (FGPS) lamination sequence (Figure 3b) was also investigated to examine if the mechanisms can be implemented without warpage, utilising the same number of layers as in the asymmetrical layup. 

The lamination sequences for the FGPA and FGPS configurations considered for this work are reported in Table 1.

## 3. Materials and Methods

### 3.1. Sample Manufacturing

**Figure 4:**
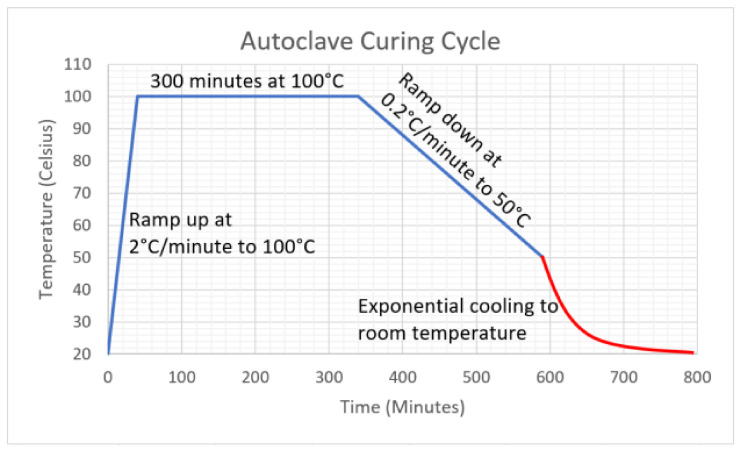
Autoclave cooling cycle.

For the experimental analysis of the FGP design, unidirectional carbon fibre prepreg “XPREG^®^ XC130” was used. Each ply was cut into 100 mm × 150 mm for the impact test and 90 mm × 270 mm for the bending samples. The lamination sequences used for the different configurations are reported in Table 1 and all the laminates were cured using the temperature cycle shown in Figure 4 in order to minimise the residual stresses [49] generated by the FGPA design (Appendix A). The final thickness of the samples is ~5.2 mm using 18 plies in total.

### 3.2. Three-Point Bending

**Figure 5:**
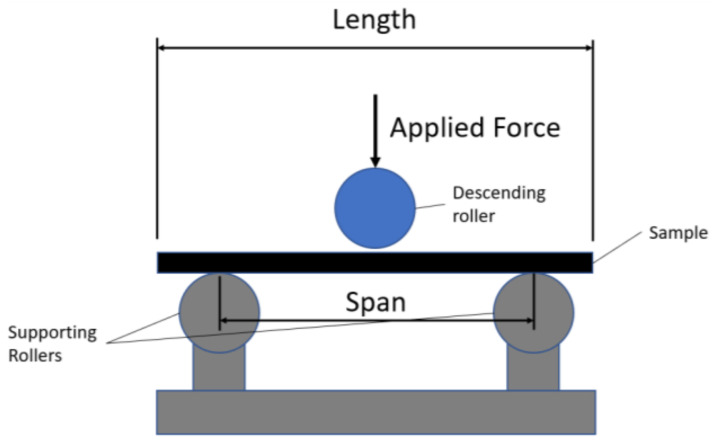
Three-point bending illustration.

The flexural properties of the different laminates were determined by three-point bending (Figure 5), following the ISO 14125:1998+A1 guidelines and using a universal Instron testing machine, model 3369, with a 50 kN load cell. Due to the quasi-static nature of impacts with a high ratio of impactor mass to equivalent structure mass [50], the static flexure test aids an understanding of the different damage mechanisms involved during the material failure. 

Supports and loading rollers of radius 5 ± 0.2 mm were used with a loading speed of 13.76 mm/min, in accordance with Equation (7), for a strain rate of 0.01 mm/min and 5.16 mm average sample thickness.

(7) $$v=\frac{\varepsilon^{\prime }L^{2}}{6h}$$

In this equation, *v* is the loading speed (mm/min), ε’ is the strain rate, *L* is the span length (mm) and *h* is the laminate thickness (mm). With a span of 206.5 mm and a 50 kN load cell used, fixed rate three-point bending tests were completed with the time, load, and deflection data logged. Flexural stress and strain data were calculated with Equations (8)–(9) considering individual sample width and thicknesses.

(8) $$\sigma_{f}=\frac{3FL}{2bh^{2}}$$

(9) $$\varepsilon_{f}=\frac{6sh}{L^{2}}$$

where *σ_f_* is the flexural stress (MPa), *F* is the applied load (N), *b* is the laminate width (mm), *ε_f_* is the flexural strain and s is the central displacement (mm). Considering the load data at flexural strains of 0.0005 and 0.0025, the flexural modulus was calculated using Equation (10):

(10) $$E_{f}=\frac{L^{3}}{4bh^{3}}\left(\frac{∆F}{∆s}\right)$$

where *E_f_* is the flexural modulus (GPa), Δ*F* is the difference in force between strains and Δ*s* is the difference in central displacement between strains. The Specific Elastic Energy (SEE), normalised by the stressed volume of the sample, was calculated as per Equation (12):

(11) $$w_{i}=\frac{\int_{0}^{x_{i}}Fdx}{dhL}$$

where *w_i_* is the instantaneous cumulative specific work done (J), *x* is the midpoint compression, *x_i_* is the instantaneous midpoint compression and *F* is the midpoint load.

### 3.3. Low Velocity Impact

**Figure 6:**
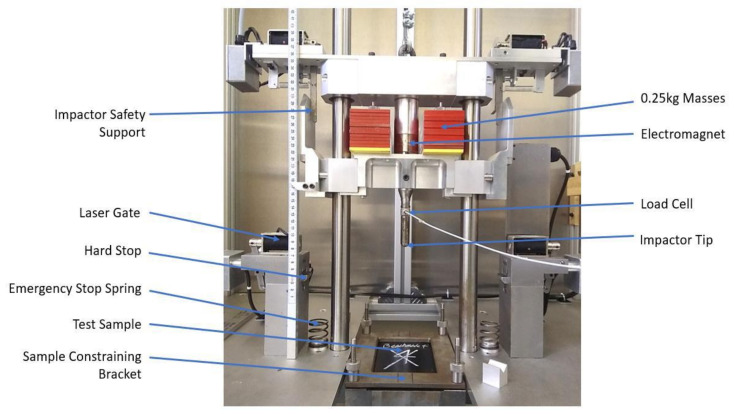
Impact rig used during the impact campaign.

Impact tests were carried out on 100 mm × 150 mm impact samples with a drop rig of adjustable impactor mass and drop height as shown in Figure 6. An oscilloscope (PICO TECHNOLOGY Picoscope) and a MATLAB code were used to collect and process the impact signal from a KISTLER loadcell. A 15 mm in diameter semi-spherical hardened steel impactor tip was used according to the ISO 6603-2:2000 standard. 

For the characterisation of impact behaviour, a selection of impact energies (15 J and 25 J) was tested for each design, with three samples per impact energy. The energy of the system was varied by the initial height of the dropped mass while holding the impactor mass constant at 8.66 kg. An anti-rebound system using two laser gates was used to avoid a second impact on the samples. After the impact, in order to correlate the energy absorption profiles with the different failure mechanisms, ultrasonic techniques were used to generate C-scan data with images reporting the damage extent and depth information over the surface plane. The phased array NDT was carried out using an ultrasonic scanner (National Instrument) with an array of 128 transducers to image a 67.3 mm wide section of each sample. 

In order to characterise the post-impact residual properties of the bioinspired laminates and estimate their residual structural integrity, Flexural After Impact (FAI) tests were carried out. Standard post-impact testing methods, including Compression After Impact (CAI) (ISO 18352:2009), commonly used to evaluate these properties, were impossible to apply for the FGPA bioinspired configuration used in this work due to significant buckling (not acceptable by the standard) [51] generated during the test related to non-zero extensional-flexural coupling terms in the stiffness matrix of the laminate. Due to the lack of standards for the FAI test, the three-point bending standard (ISO 14125:1998+A1) and previous research works [52,53] were used as guidelines. Loading and support rollers used in this experimental campaign were 25 mm in diameter; the span between the support rollers was set to 100 mm while the crosshead speed of the loading roller was 4.5 mm/min. The impacted sample was inserted into the machine and the load was applied until a drop of 60% of the maximum recorded load was identified. The post-impact residual energy *W_residual_* (J) involved in the process was calculated using Equation (12):

(12) $$W_{residual}=\int_{0}^{d_{max}}Fdx$$

where *d_ma_*_x_ is the displacement reached when the force drops to 60% of the maximum force recorded. 

## 4. Results and Discussion

### 4.1. Flexural Tests

**Figure 7:**
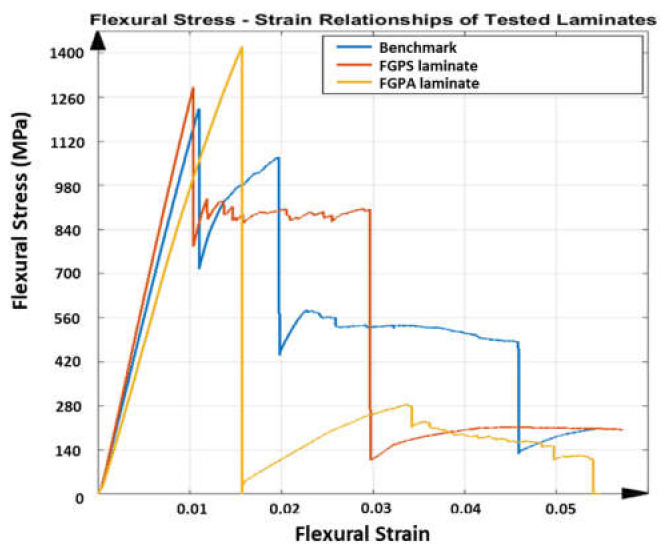
Flexural stress–strain curves for the benchmark, symmetric (FGPS) and asymmetric (FGPA) configurations.

The flexural stress–strain results of the benchmark, FGPS and FGPA samples collected using three-point bending testing are shown in Figure 7.

**Figure 8:**
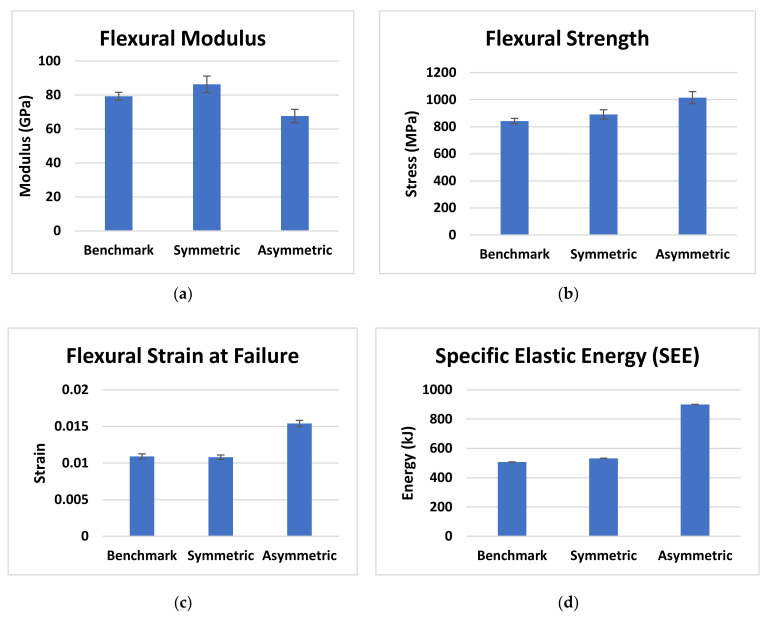
Bar plot results for the benchmark, FGPS and FGPA configurations: (**a**) flexural modulus; (**b**) flexural strength and (**c**) flexural strain at maximum force value; and (**d**) Specific Elastic Energy (SEE).

Flexural data are reported in Figure 8 where the flexural modulus, flexural strength, flexural strain at failure and SEE stored during the tests are shown. It is important to notice that the specific elastic energy values are calculated considering the maximum flexural force and the corresponding strain value that represents the flexural strain at failure. Table 2 reports the mean and standard deviation of the flexural data benchmark, FGPS and FGPA configurations.

**Figure 9:**
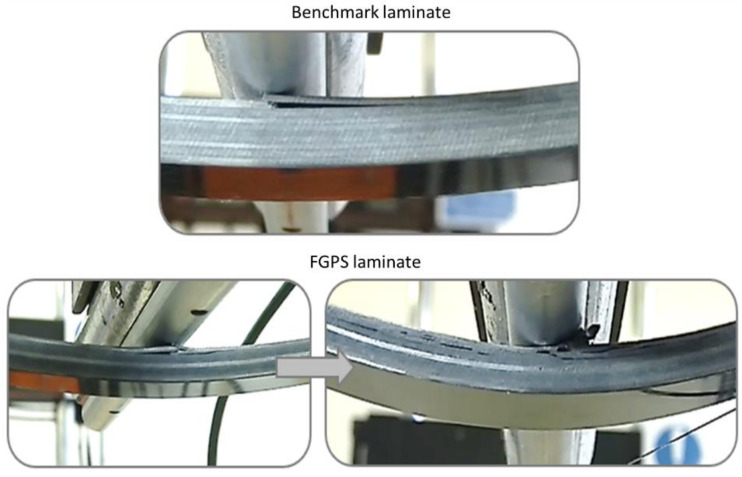
Initial damage propagation of the benchmark and FGPS laminates.

As reported in Figure 7, the FGPS configuration shows an initial elastic behaviour similar to the benchmark with a slight variation in the flexural modulus (Figure 8a), flexural strength (Figure 8b) and elastic energy stored (Figure 8d) of +9%, +6% and +5%, respectively. One can notice that the flexural stress for the FGPS samples remains approximately constant with strain after the initial load drop given by the failure of the top layer and visible damage below the top 0° plies, as shown in Figure 9.

This is divergent from the benchmark behaviour, which experienced a gradual reduction in flexural stress, increasing the applied strain. This behaviour can be attributed to the pure delamination case of the damage propagation in the benchmark sample, contrasting the more complex evolution of damage downwards in the FGPS laminate. The propagation of this damage through adjacent plies is affected by the higher interlaminar strength provided by the small angle difference between the two plies [17], which eases the crack jumping from layer to layer [39] and without the generation of wide delaminated areas. Once initiated, the crack progressively propagates throughout the laminate’s thickness following a tortuous path that dissipates a higher amount of energy, preventing flexural stress from increasing with strain and generating a plateau region in the stress–strain plot. Due to the reduced stiffness resulting from damage in the composites [9], the brittleness of the FGPS is reduced and causes the flexural strain at the secondary load drop to significantly increase. 

**Figure 10:**
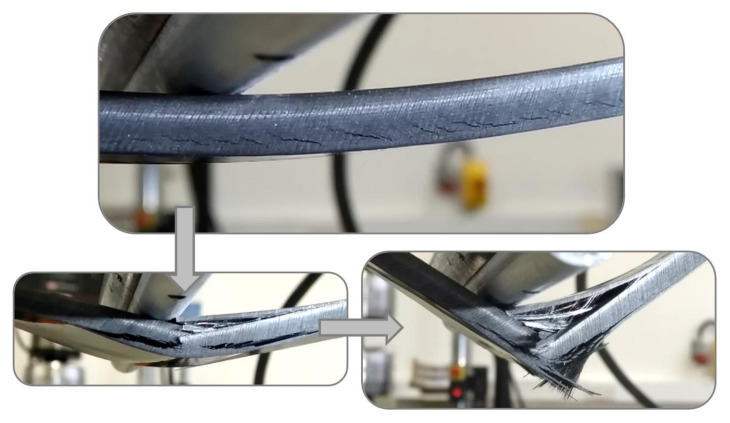
Damage evolution in FGPA laminates: sub-critical damage (top image) and critical damage (bottom images).

Analysing the results from FGPA configuration (Figure 7 and Figure 8), it is possible to observe a higher maximum flexural stress (+21%) and strain (+41%) than the benchmark one at the first load drop. This behaviour causes a greater amount of energy to be stored in the FGPA laminate (+78% compared to benchmark) before damage, which improves the damage resistance of this configuration by requiring a greater energy to initiate a critical damage. Indeed, FGPA structure initiates damage with a significantly different mechanism than the other tested laminates. This is due to the functionally graded angle used to manufacture the sample as the smaller the pitch angle, the smoother the crack propagation across the different plies, leading to higher damage tolerance and reduced delamination extent [19]. Moreover, due to the small variable angle between the plies, the stiffness of the laminate varies across the thickness, as described in Section 2.1. Therefore, in the area where a small angle pitch is used, the lower laminate stiffness allows to store a higher amount of elastic energy in the material during deformation, since virtually no delamination is generated during the failure initiation and propagation due to the activation of the crack twisting [18]. On the other hand, in the area where the ply angle is greater, a higher value of stiffness is reported that balances the effect of the small initial ply angle on the global stiffness of the laminate but, at the same time, induces a higher sensitivity towards the generation of non-critical damage. This is confirmed by observing the images in Figure 10 where the failure mechanisms are shown, where subcritical stable damage is visible before failure. 

The cause of this subcritical damage in the tensile portion is the increasing angle ply that allows the generation of a higher amount of interlaminar damage. However, this subcritical damage is stable and has no tendency in degenerating into an unstable critical one as found, instead, in the benchmark case. In addition, since the failure of the laminate is initiated and dominated by the crack twisting mechanism in the compressive portion, this stable damage generated within the tensile portion has not only a marginal detrimental effect on the mechanical properties of the laminate but also helps the system to dissipate a higher amount of energy, enabling the system to reach a large strain without the critical failure of the interested layers [16]. Thus, even though a reduced stiffness (−15% compared to benchmark) is associated with the presence of this sub-critical damage, comparing these configurations with the benchmark and FGPS, a higher maximum flexural strength in the elastic portion of the curve is identified at a higher strain at failure values. 

### 4.2. Impact Tests

**Figure 11:**
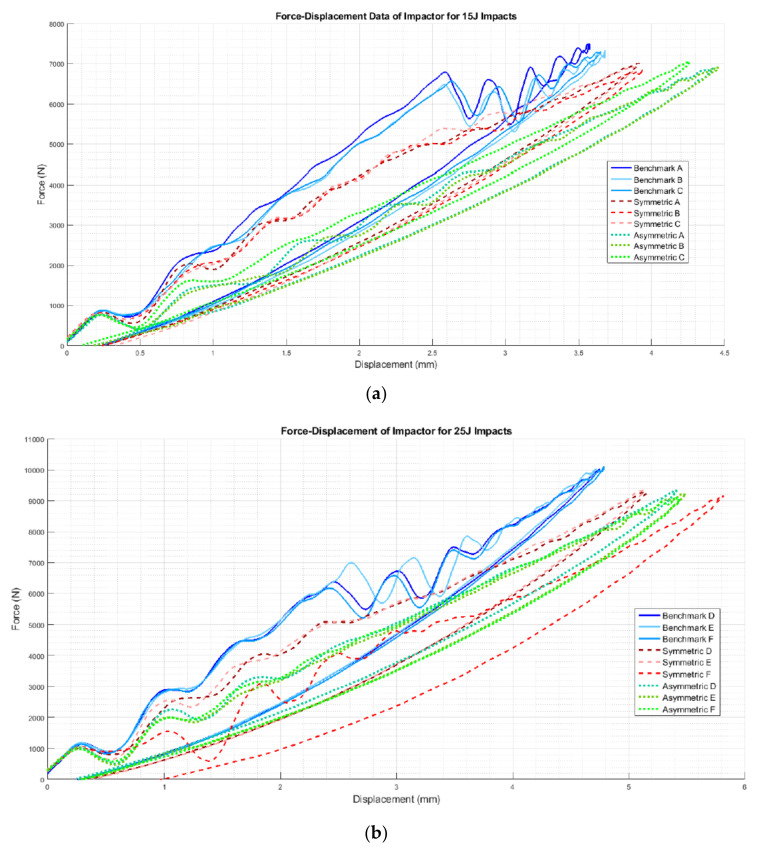
Force–displacement curves for the benchmark, symmetric and asymmetric configurations.

**Figure 12:**
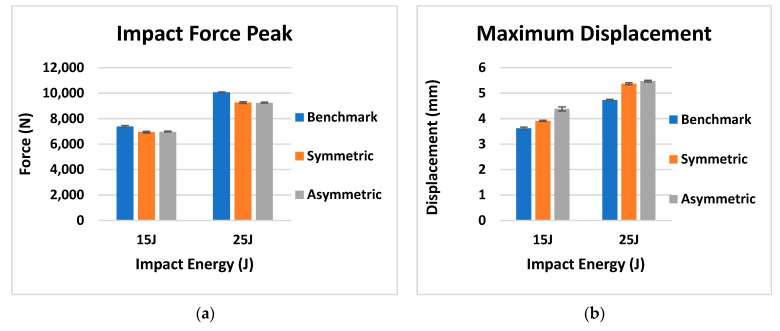

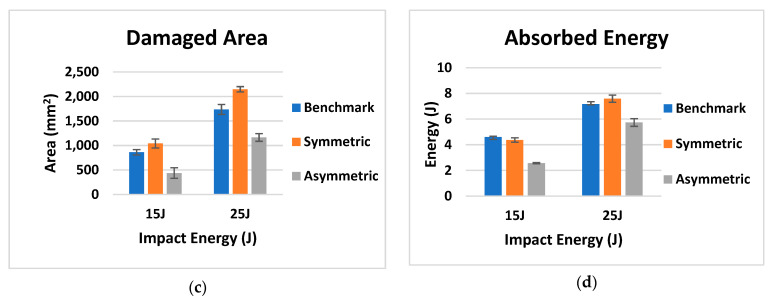
Bar plot with the mean and standard deviation of the (**a**) impact force peak, (**b**) maximum displacement, (**c**) damaged area and (**d**) absorbed energy for the benchmark, symmetric (FGPS) and asymmetric (FGPA) configurations.

Force–displacement data from the 15 and 25 J impacts is shown in Figure 11 while the impact results, including the mean and standard deviation of the impact force, maximum displacement, damaged area and absorbed energy, are reported in Figure 12. In Table 3, the mean and standard deviation of the impact results for the different configurations are reported.

**Figure 13:**
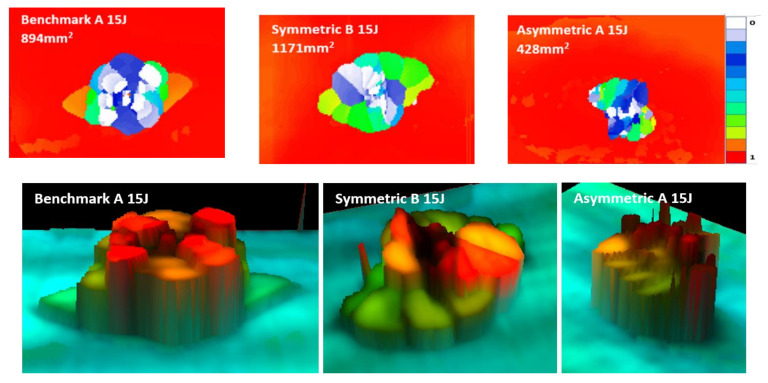
Time-of-flight C-scans and 3D images of the 15 J impact samples for the benchmark, symmetric (FGPS) and asymmetric (FGPA) configuration.

Considering the impact curves at 15 J in Figure 11a, it is possible to observe the impact force initially increasing with displacement for all the designs. A slight reduction in peak impact force for the FGPS (−6%) and FGPA (−6%) laminates in comparison with the benchmark is reported, showing no significant load drops, as was observed in the benchmark, between ~2 and 4 mm, which are typical in laminate damage mechanisms. Moreover, as shown in Table 2 and explained for the flexural tests in Figure 8, higher maximum displacement values are shown for the FGPS (+8%) and FGPA (+21%) configurations when compared to the benchmark due to the functionally graded characteristics of in the lamination sequence due to the use of an FGP angle ply that allows a more uniform impact energy distribution along through the thickness direction of the laminate [54]. Comparing these impact curves with the flexural ones (Figure 7), it is possible to notice a different trend in terms of mechanical stiffness (slope of the curves). This is explained considering the contribution to bending and shear stress on mechanical response of the material for the two different experimental cases. In the flexural case (three-point bending condition), the standard guidelines were followed by setting the width of the beam to a certain value (15 mm) and its span/thickness to 40. This allows to neglect the major shear effects during the experimental tests and evaluate the mechanical response of pure bending [55]. This translates into a higher stiffness, as recorded for the FGPS and FGPA configurations in comparison with the benchmark, since a higher number of plies close to the 0° direction is used in their stacking-up sequence. On the contrary, no pure bending condition can be achieved in the impact case (low velocity impact condition) since a plate geometry (150 mm × 100 mm) is used as described by the standard guidelines leading to a shear-dominant mechanical response [45]. Consequently, the higher number of plies oriented along 0° has no beneficial effects for the FGPS and FGPA configurations and a lower stiffness is recorded in comparison with the benchmark. The energy absorbed by the samples during impact is reported in Figure 12d, where similar values between the FGPS and benchmark laminates are shown. The FGPA laminates, instead, reported a reduced energy absorption when compared to the benchmark (−44%), which can be correlated to a reduction in damaged area. Thus, in order to investigate the extent and distribution of internal damage of the FGPS and FGPA configurations generated as results of the impact loading, a phased array ultrasound technique was used. The time-of-flight C-scans of 15 J samples are shown in Figure 13 using a normalised reflection depth scaling (colour map—16 bit) from 0 (white) to 1 (red).

**Figure 14:**
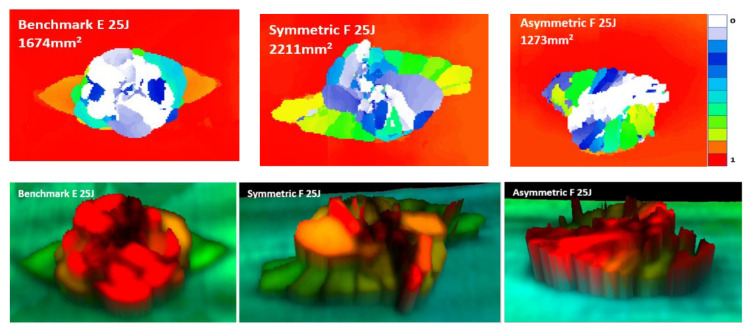
Time-of-flight C-scans and 3D images of the 25 J impact samples for the benchmark, symmetric (FGPS) and asymmetric (FGPA) configuration.

Figure 13 shows that the benchmark laminate experiences peanut-shaped damage [1], whilst the FGPS and FGPA laminates display alternative damage mechanisms. In the same figure, three-dimensional images based on damage depth are shown in order to visualise the shapes of the damage within laminates. As it is possible to see from these images, damage in the FGPS and FGPA laminates follows a twisting crack, which acts as a toughening mechanism [17] and reduces the area of delamination. This is due to the ability of helicoidal structures to initiate and propagate the crack along a specific path given by the ply angle. Indeed, as already shown for the flexural data discussion, the crack path in FGP helicoidal laminates is forced to twist following the ply angle that varies along the thickness of the laminate, accumulating sub-critical stable damage within the laminate’s body, as was also reported by [16] in a similar case study. This is a less detrimental damage topology than delamination, which is considered an unstable, critical one. The damaged area of the 15 J impact samples is shown in Figure 12c. FGPS laminates propagate damage over a larger area than the benchmark (+21%), whilst the FGPA laminates have a greatly reduced damaged area (−44%). This is due to the variable stiffness across the thickness of the FGPA design, also observed during the flexural tests, which enables the laminate to store a higher amount of elastic energy during the dynamic event and consequently to reduce the amount that is dissipated via the creation of new surfaces [56]. Figure 11b shows the reduced peak force for the helicoidal laminates at the 25 J impacts when compared to the benchmark ones. In particular, the FGPS configuration shows a variation in force peak and maximum displacement of −8% and +14%, respectively, compared to the benchmark, while FGPA illustrates a force peak reduction of −8% and a maximum displacement variation of +16%. Slight load drops are visible in both the helicoidal configurations near the peak force at this energy, which indicates a reduced stiffness from the structural damage. Figure 12d shows the absorbed energy of the FGPS laminates, which slightly exceeds the benchmark at the 25 J impact energy (+6%), whilst the FGPA laminate is characterised by reduced energy absorption. The crack twisting mechanism contributing to energy absorption are visible in the ultrasonic C-scans and 3D damage images in Figure 14, where it is possible to observe how the crack is able to rotate during propagation according to the ply angle used.

As it is possible to see from the images, the different configurations show similar damage shapes between the 15 J (Figure 13) and 25 J impacts, with larger damage propagation for the 25 J case. 3D images also show twisting cracks in both helicoidal laminate designs, with the propagation of the crack front oriented according to the local ply angle, indicating that these designs successfully exploited this toughening mechanism, minimising the generation of delaminated areas. Analysing the damaged area reported in Figure 12c, it is possible to notice a similar trend in the extent of the damaged area between the 15 J and 25 J cases, with an increase in the FGPS laminates and reduction in the FGPA one when compared to the benchmark. 

**Figure 15:**
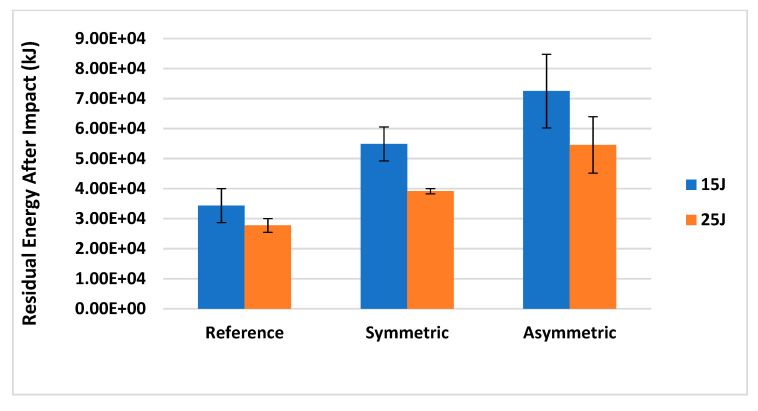
Results of the FAI tests.

In order to confirm the ability of these bioinspired structures to improve the damage tolerance when introduced into a laminated composite, Flexural After Impact (FAI) tests were carried out on the impacted samples and the output results are reported in Figure 15 and Table 4.

Analysing the data in this figure, it is clear that all the configurations impacted at 15 J show a higher post-impact residual energy than the ones impacted at 25 J, since a greater damaged area is generated within the laminate. A significant difference is identified between the bioinspired and traditional configurations, reporting, at 15 J, an increase in post-impact residual energy of +60% and +111% for FGPS and FGPA when compared with the benchmark configuration. Similarly, at 25 J, the post-impact energy residual is +41% (FGPS) and +97% (FGPA) higher than the one of the benchmark, confirming the ability of these bioinspired structures to promote the propagation of twisted cracks within the laminate, generating subcritical damage and minimising the number of delaminated areas within the part. Thus, since the structure integrity is less compromised, the two bioinspired structures can store a higher amount of energy than traditional ones when transversally loaded after an impact event. Comparing the results obtained between the bioinspired configurations, instead, the FGPA configuration shows a higher residual energy than the FGPS one since a smaller damaged area is identified within the laminate’s body. This can be attributed to the efficiency of this structure in maximising the benefits of the crack twisting mechanisms given by the smoother variable stiffness across the laminate’s thickness and the reduced extent of delaminated areas generated during the impact event.

## 5. Conclusions

The ability of helicoidal laminates featuring a novel Functionally Graded (FGP) layup sequence in improving the impact performance of CFRP laminates has been explored via static three-point bending and impact testing. Both symmetrical (initial/final angle: 5/40°) and asymmetric (initial/final angle: 1.2/20°) helicoidal designs (FGPS and FGPA, respectively) demonstrated enhanced twisting-crack damage mechanisms, which mimics in detail the behaviour of biological structures, including the dactyl club of the mantis shrimp. The successful replication and activation of this unique failure mechanisms was confirmed by photographic evidence, revealing extensive twisted cracking in the matrix in the bending samples. 

The Functionally Graded Symmetric (FGPS) helicoidal laminate shows a slight improvement in flexural strength (+6%) and modulus (+9%), as per the three-point bending tests. On the other hand, no significant load drops and a reduced peak impact force (6–8%) were identified from the impact results for both the impact energies considered during impact testing (15 J and 25 J). However, the reduction in damaged area (~21–24%) and increase in post-impact residual energy (41–60%) in comparison with the traditional laminates used as benchmarks indicates this design is successful in fully exploiting the helicoidal architecture in improving toughness. This is due to the crack twisting failure mechanism that dissipates large quantities of impact energy in creating stable matrix cracks instead of unstable delaminated areas, as shown in the post-impact phased array testing. 

The Functionally Graded Asymmetric (FGPA) helicoidal laminate instead shows a greatly reduced impact damaged area (−33–49%) and absorbed energy (−20–44%), but a significant increase in post-impact residual energy (91–111%). In addition, this structure shows in flexural loading conditions greater mechanical strength (21%) and elastic strain (+41%). The reason for this improved flexural and impact behaviour can be found in the coupling effect between crack twisting and variable stiffness along the thickness of the laminate that allows for storing a higher amount of impact energy elastically and efficiently dissipating the excess via sub-critical twisted cracks that reduces the delaminated areas, increasing the residual mechanical properties of the part. 

Based on these results, an FGPA configuration can express its full potential in applications where a superior impact resistance and damage tolerance are mandatory for the safety and reliability of the global structure.

An FGPS configuration, instead, can be successfully utilised as an alternative to the FGPA configuration in order to improve the impact response of the composite material in all those applications where geometrical stability and damage tolerance are required. 

## Figures and Tables

**Table 1:** Bioinspired composite designs based on the mantis shrimp dactyl club.

Lay-Up Title	Ply Structure
Benchmark	[0/0/+45/−45/90/0/+45/−45/90]_s_
FGPS (*c* = 1.2)	[0/5/15/30/50/75/105/140/180]_s_
FGPA (*c* = 5)	[0/1.2/3.5/7.1/11.8/17.7/24.7/32.9/42.4/52.9/64.7/77.6/91.8/107.1/123.5/141.2/160/180]

**Table 2:** Mean and standard deviation for the flexural data of the benchmark, symmetric (FGPS) and asymmetric (FGPA) configurations.

Design	Flexural Modulus (GPa)	% Variation	Flexural Strength (MPa)	% Variation	Flexural Strain (Maximum Load)	% Variation	SEE (kJ/m^3^)	% Variation
Benchmark	79.2 ± 2.32	-	842 ± 19.56	-	0.0109 ± 0.00035	-	507 ± 25	-
FGPS	86.3 ± 4.88	+9%	891 ± 34.8	+6%	0.0154 ± 0.00030	−1%	532 ± 25	+5%
FGPA	67.6 ± 3.91	−15%	1015 ± 44.37	+21%	0.0154 ± 0.00042	+41%	900 ± 63	+78%

**Table 3:** Mean and standard deviation of the impact results for the benchmark, symmetric (FGPS) and asymmetric (FGPA) configurations.

Configuration	Impact Energy (J)	Impact Force Peak (N)	% Variation	Maximum Displacement (mm)	% Variation	Damaged Area (mm^2^)	% Variation	Absorbed Energy (J)	% Variation
Benchmark	15	7377.08 ± 88.4	-	3.61 ± 0.052	-	859.97 ± 54.74	-	4.60 ± 0.057	-
Symmetric	15	6934.79 ± 64.54	−6%	3.91 ± 0.022	+8%	1041.12 ± 92.19	+21%	4.36 ± 0.172	−5%
Asymmetric	15	6955.12 ± 62.43	−6%	4.37 ± 0.080	+21%	437.24 ± 106.54	−49%	2.56 ± 0.042	−44%
Benchmark	25	10,059.44 ± 44.58	-	4.72 ± 0.033	-	1734.86 ± 100.82	-	7.17 ± 0.1857	-
Symmetric	25	9259.95 ± 74.99	−8%	5.37 ± 0.390	+14%	2147.79 ± 54.24	+24%	7.59 ± 0.2763	+6%
Asymmetric	25	9229.94 ± 73.07	−8%	5.47 ± 0.037	+16%	1163.69 ± 79.05	−33%	5.73 ± 0.2970	−20%

**Table 4:** Data for the FAI tests reporting the mean of the residual energy (kJ) and relative variation from the benchmark for each configuration (FGPS and FGPA).

Residual Energy (kJ)	15 J	% Variation	25 J	% Variation	Standard Deviation	15 J	25 J
Benchmark	3.43 × 10^4^	0%	2.77 × 10^4^	0%	Benchmark	5.66 × 10^3^	2.30 × 10^3^
FGPS	5.49 × 10^4^	60%	3.91 × 10^4^	41%	FGPS	5.65 × 10^3^	8.68 × 10^2^
FGPA	7.25 × 10^4^	111%	5.45 × 10^4^	97%	FGPA	1.23 × 10^4^	9.41 × 10^3^

**Figure A1:**
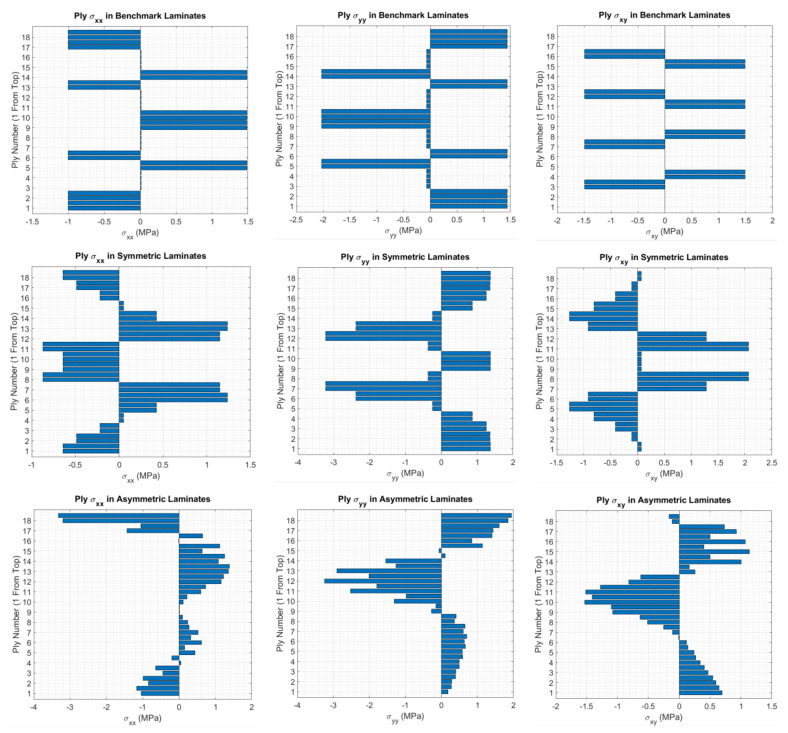
Laminate residual thermal stresses.

## Data Availability

Data sharing is not applicable for this article.

## Appendix A. Thermal Warpage

In order to analyse the thermal behaviour of the helicoidal configurations and understand the design limitations given by the potential residual thermal stresses, analytical methods were used. Classical laminate theory [57] can be applied in a plane stress state, which first requires calculation of the local coordinate lamina stiffness matrix given by Equation (A1).

(A1) $$\left[C\right]=\left[\begin{array}{ccc} C_{11} & C_{12} & 0\\ C_{21} & C_{22} & 0\\ 0 & 0 & C_{66} \end{array}\right]$$

with:

(A2) $$C_{11}=\frac{E_{11}}{1-v_{12}v_{21}}$$

(A3) $$C_{22}=\frac{E_{22}}{1-v_{12}v_{21}}$$

(A4) $$C_{12}=C_{21}=\frac{v_{12}E_{22}}{1-v_{12}v_{21}}=\frac{v_{21}E_{11}}{1-v_{12}v_{21}}$$

(A5) $$C_{66}=G_{12}$$

where *[C]* is the local coordinate lamina stiffness matrix, *E*_11_ is the longitudinal Young’s Modulus of the lamina (~135 GPa), *E*_22_ is the transverse Young’s Modulus (~8.5 GPa), *v*_12_ is the major Poisson’s Ratio (~0.33), *v*_21_ is the minor Poisson’s Ratio (~0.021) and *G*_12_ is the Shear Modulus (~5 GPa). Using the transformation matrix in Equation (A1), Equation (A6) is used to obtain the local stiffness matrix of each lamina to the global coordinate system.

(A6) $$\left[T\right]=\left[\begin{array}{ccc} \cos^{2}\vartheta & \sin^{2}\vartheta & \frac{1}{2}\sin2\vartheta \\ \sin^{2}\vartheta & \cos^{2}\vartheta & -\frac{1}{2}\sin2\vartheta \\ -\sin2\vartheta & \sin2\vartheta & \cos2\vartheta \end{array}\right]\;$$

(A7) $$\left[Q\right]={\left[T\right]}^{t}\;\left[C\right]\;\left[T\right]$$

where [*T*] is the transformation matrix, *ϑ* is the lamina rotation angle and [*Q*] is the lamina stiffness matrix in global coordinates. Calculating the [*Q*] matrices for all laminae enables computation of the behaviour-defining [*A*], [*B*] and [*D*] matrices from Equations (14)–(16).

(A8) $$\left[A\right]=\overset{N}{\underset{k=1}{\sum }}\left[Q_{k}\right]\;t_{k}$$

(A9) $$\left[B\right]=\overset{N}{\underset{k=1}{\sum }}\left[Q_{k}\right]\;t_{k}\;\bar{z_{k}}$$

(A10) $$\left[D\right]=\overset{N}{\underset{k=1}{\sum }}\left[Q_{k}\right]\;\left(t_{k}\;{\bar{z_{k}}}^{2}+\frac{t_{k}^{3}}{12}\right)$$

where [*A*] is the laminate membrane stiffness matrix, [*B*] is the laminate coupling matrix, [*D*] is the laminate bending stiffness matrix, t is the thickness of a single ply and $\bar{z}$ is the mean average height of each ply from the laminate midplane.

Table A1 data were calculated for each configuration using Equations (A8)–(10) and considering the lamination sequences reported in Table 1.

**Table A1:** Composite laminate matrices.

Benchmark	
[A] Matrix:	$\left[\begin{array}{ccc} 341.32 & 82.75 & 0\\ 82.75 & 268.26 & 0\\ 0 & 0 & 93.98 \end{array}\right]$kN/mm
[B] Matrix:	$\left[\begin{array}{ccc} 0 & 0 & 0\\ 0 & 0 & 0\\ 0 & 0 & 0 \end{array}\right]$kN
[D] Matrix:	$\left[\begin{array}{ccc} 1075.86 & 146.39 & 24.04\\ 146.39 & 352.70 & 24.04\\ 24.04 & 24.04 & 171.34 \end{array}\right]$kNmm
**Functionally Graded Helicoidal Symmetric**	
[A] Matrix:	$\left[\begin{array}{ccc} 409.49 & 73.71 & 39.97\\ 73.71 & 218.16 & 16.28\\ 39.97 & 16.28 & 84.94 \end{array}\right]$kN/mm
[B] Matrix:	$\left[\begin{array}{ccc} 0 & 0 & 0\\ 0 & 0 & 0\\ 0 & 0 & 0 \end{array}\right]$kN
[D] Matrix:	$\left[\begin{array}{ccc} 1224.64 & 118.85 & 165.64\\ 118.85 & 259.01 & 68.57\\ 165.64 & 68.57 & 143.79 \end{array}\right]$kNmm
**Functionally Graded Helicoidal Asymmetric**	
[A] Matrix:	$\left[\begin{array}{ccc} 399.12 & 75.98 & 38.46\\ 75.98 & 223.99 & 17.16\\ 38.46 & 17.16 & 87.21 \end{array}\right]$kN/mm
[B] Matrix:	$\left[\begin{array}{ccc} 207.27 & -28.52 & 93.77\\ -28.52 & -150.22 & 44.22\\ 93.77 & 44.22 & -28.52 \end{array}\right]$kN
[D] Matrix:	$\left[\begin{array}{ccc} 1213.94 & 112.65 & -47.60\\ 112.65 & 282.11 & -65.66\\ -47.60 & -65.66 & 137.59 \end{array}\right]$kNmm

The thermal stresses induced from cooling after the elevated temperature autoclave cycle are calculated per Equation (A11).

(A11) $${\left\{\sigma \right\}}^{k}={\left[C\right]}^{k}\;\left({\left\{\varepsilon \right\}}^{k}-{\left\{\alpha_{T}\right\}}^{k}∆T\right)$$

where *σ*^(*k*)^ is local stress vector of kth ply, [*C*] is local stiffness matrix of the kth ply, *ε*^(*k*)^ is the local strain vector of the kth ply, *α*_*T*_^(*k*)^ is the thermal expansion coefficient vector of the kth ply and Δ*T* is the change in temperature after curing.

Applying Equation (A11) resulted in residual thermal stresses described in Figure A1.

As can be seen from this figure, the non-zero [B] matrix in the FGPA laminates causes in-plane to out-of-plane coupling of forces and deformations, leading to thermal-induced warpage and twist. Consequently, the thermal residual stress on FGPA samples requires a custom cure cycle to be minimised with a low curing temperature and cool rate.

FGPS configuration, instead, was designed using a greater variable ply angle and a symmetrical layup obtaining a zero [B] matrix that generates no thermal residual stress on the laminate geometry during the cure (Figure A1).
